# A review of the mechanisms underlying selected comorbidities in Alzheimer’s disease

**DOI:** 10.1007/s43440-021-00293-5

**Published:** 2021-06-13

**Authors:** Karolina Maciejewska, Kamila Czarnecka, Paweł Szymański

**Affiliations:** 1 Department of Pharmaceutical Chemistry, Drug Analyses and Radiopharmacy, Faculty of Pharmacy, Medical University of Lodz, Muszynskiego 1, 90-151 Lodz, Poland; 2 Department of Radiobiology and Radiation Protection, Military Institute of Hygiene and Epidemiology, 4 Kozielska St, 01-163 Warsaw, Poland

**Keywords:** Alzheimer's disease, Comorbidities, Depression, Diabetes mellitus, Down syndrome, Hypercholesterolemia

## Abstract

Alzheimer's disease (AD) is a progressive neurodegenerative disorder of the central nervous system (CNS) leading to mental deterioration and devastation, and eventually a fatal outcome. AD affects mostly the elderly. AD is frequently accompanied by hypercholesterolemia, hypertension, atherosclerosis, and diabetes mellitus, and these are significant risk factors of AD. Other conditions triggered by the progression of AD include psychosis, sleep disorders, epilepsy, and depression. One important comorbidity is Down’s syndrome, which directly contributes to the severity and rapid progression of AD. The development of new therapeutic strategies for AD includes the repurposing of drugs currently used for the treatment of comorbidities. A better understanding of the influence of comorbidities on the pathogenesis of AD, and the medications used in its treatment, might allow better control of disease progression, and more effective pharmacotherapy.

**Graphic abstract:**

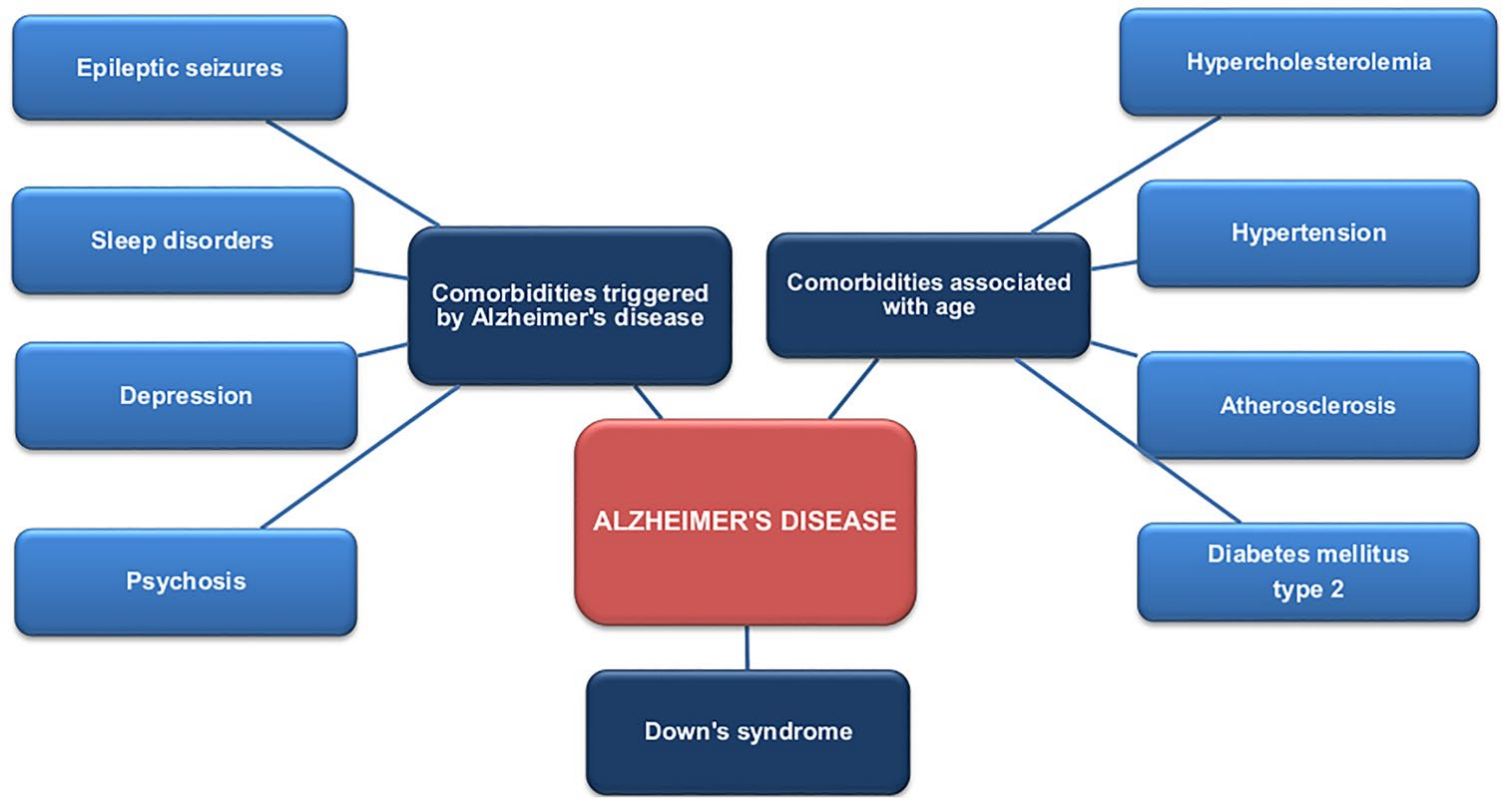

## Introduction

The most common type of senile dementia is Alzheimer' s disease (AD). This incurable neurodegenerative disorder, characterized by a progressive decline of cognitive function, eventually leads to death. It mostly affects people above the age of 65 years [1]. Official reports indicate that 50 million people worldwide are affected by AD and other types of dementia, and this number is expected to triple by 2050 [2]. AD is now most common in Western Europe and the US; however, a rapid increase in morbidity is predicted in low and middle-income countries in the near future, especially African countries and India [2, 3].

According to the novel guidelines of the National Institute on Aging and Alzheimer's Association (NIA-AA) [4], the term *Alzheimer's disease* refers to patients with diagnosed Aβ plaques and tau deposits, detected in vivo by abnormal biomarkers of Aβ, and pathologic tau or postmortem examination. In contrast, the recommended terminology for patients with clinical symptoms of dementia, but not proved by any biomarkers characteristic of AD is *Alzheimer's clinical syndrome*, while the term for the presence of AD-specific biomarkers is *Alzheimer's continuum*. The NIA-AA also developed the concept of *Alzheimer's pathologic change*, which refers to an early stage of the Alzheimer's continuum, defined in vivo by an abnormal Aβ biomarker without a pathologic tau biomarker [4].

The main risk factor for AD is advanced age [5]. It is believed that the development of the disease is strongly driven by Down’s syndrome, diabetes mellitus type 2, heart disease, hypertension, obesity, and inflammation processes [1]. Genetic mutations appears to be a primary cause of early-onset AD, especially the familial form, where the first symptoms occur before 65 years of age. Of these, autosomal dominant inherited gene mutations, such as amyloid precursor protein (*APP*), presenilin 1 (*PSEN1*), and presenilin 2 (*PSEN2*), which promote Aβ production, appear to have the greatest impact on AD development [6].

The presence of variant 4 of apolipoprotein E is a well-documented risk factor for late-onset AD, and one that constitutes nearly 25–50% of all AD cases [6, 7]. Double allele 4 ApoE increases the risk of AD 12-fold in women and tenfold in men [8]. In addition, the recent studies indicate that smoking, pesticides, and heavy metal pollution may also have a general impact on human mental health [5].

The development AD is complicated. Many hypotheses of AD pathomechanism have been proposed, with the three most widely documented being dysfunction of cholinergic neurotransmission, aggregation of β-amyloid protein (β-amyloid cascade), and hyperphosphorylation of tau protein [9, 10]. The cholinergic hypothesis suggests that AD may arise in response to insufficient acetylcholine production and the decline of neuronal sensitivity to acetylcholine [9]. Following on from this hypothesis, reversible acetylcholinesterase inhibitors are widely used in the therapy of AD, with a therapeutic effect being achieved in mild to moderate stages [9]. Nowadays, three reversible AChE inhibitors are approved for the use in the therapy of AD: donepezil, rivastigmine, and galantamine [11–16].

AD can also be treated with memantine, a noncompetitive antagonist of the *N*-methyl-D-aspartate (NMDA) receptor [17]. It has been reported that an excessive level of glutamate in synapses is linked with cytotoxicity and participates in pathological processes characteristic for AD. Memantine is often used in therapy of moderate to severe stages of AD [17]. Another interesting possible treatment is aducanumab, a human monoclonal antibody acting against the epitope found on Aβ. Very promising results have been obtained after the third phase of clinical trials (NCT02477800) based on a group of people with mild AD. If the FDA approved the drug in June 2021, aducanumab would be the first to act directly on Aβ [18, 19].

The second hypothesis, the β-amyloid cascade, is regarded as the most common cause of AD [20]. It has been reported that the development of AD is associated with the deposition of plaques in grey matter caused by β-amyloid aggregation and subsequent polymerization to insoluble fibrils [21]. This β-amyloid plaque formation has been attributed to the activity of the Aβ40 and Aβ42 because they are more susceptible to conformation changes and fibrillogenesis [20]. Since 1992, when the *amyloid hypothesis* was formulated, all efforts to create successful antiamyloid therapy were disappointing. Nevertheless, Aβ remains a known feature of AD, and antiamyloid therapy is one of the main future strategies to combat AD [22–25]. One promising strategy involves the use of γ-secretase and β-secretase inhibitors and modulators. Several BACE-1 inhibitors (verubecestat, atabecestat, elenbecestat, umibecestat, and lanabecestat), and γ-secretase inhibitors (semagacestat and avagacestat) and modulators (tarenflurbil) have been developed recently and have reached phase II/III clinical trials [22]. Alternatively, multitarget therapeutic strategies have been proposed in which molecules act on several pathomechanisms simultaneously; two such groups of molecules are dual AChEI/BACE-1, and AChEI/GSK-3β inhibitors, and these are being extensively studied [25]. Another promising strategy is the use of monoclonal antibodies to develop active and passive immunization against Aβ42 [12, 20, 21, 26–29].

The pathogenesis of AD is associated with the formation of neurofibrillary tangles (NFTs) [30]. Hyperphosphorylation of the tau protein leads to microtubule destabilization and the dissociation of tau from tubulin. The dissociated and hyperphosphorylated tau aggregates and forms neurotoxic tangles, which are deposited in the intracellular neuronal space. These NFTs destabilize the microtubules and eventually lead to neuronal cell death. Therefore, one therapeutic goal may be to target excessive tau hyperphosphorylation or aggregation processes [20, 30, 31].

AD is associated with multiple comorbidities and medical conditions [32, 33]. Some are associated with advanced age, while others or are triggered by various pathological conditions, such as hypercholesterolemia, hypertension, diabetes mellitus type 2, atherosclerosis, psychosis, depression, epilepsy, sleep disturbance, and Down’s syndrome [33]. Some conditions observed in AD patients such as pneumonia, urinary incontinence, osteoarthritis, and osteoporosis, and visual and hearing impairment, result from the progression of neurodegeneration [34].

This paper reviews the most common comorbidities known to be correlated with advanced age and triggered by AD. Its aim is to clarify and summarize the relationship between the comorbidities and the pathogenesis of AD. It also presents some of the medications used in the treatment of comorbidities which may be repurposed into new therapeutic strategies for AD; in addition, the paper summarizes their influence on the cognitive and behavioral symptoms of AD based on the novel in vitro/in vivo studies and clinical trials [34].

## Comorbidities in Alzheimer’s disease

**Fig. 1:**
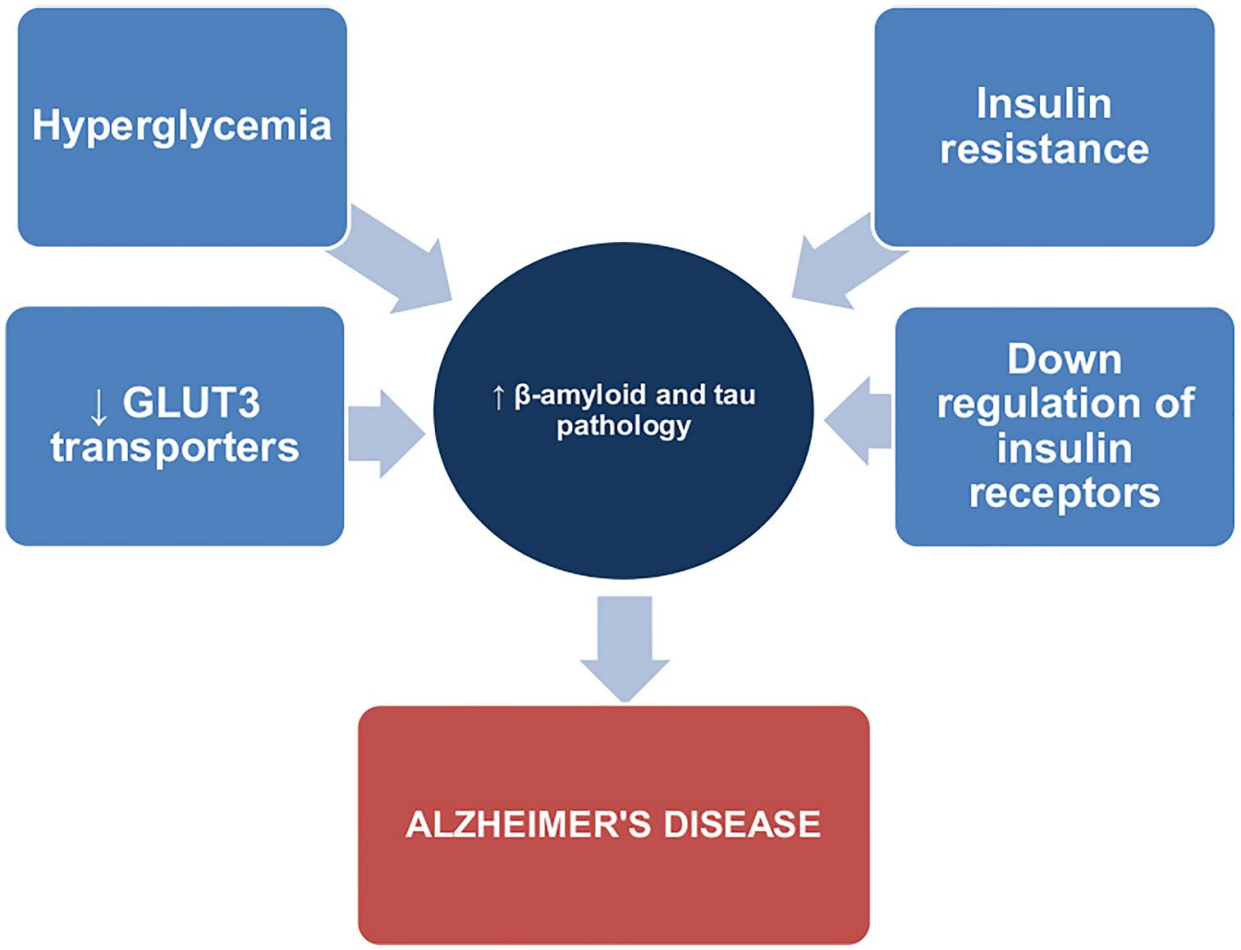
Influence of diabetes mellitus type 2 on the pathogenesis of Alzheimer's disease

Neurodegenerative disorders, such as AD are associated with multiple diseases. The comorbidities affecting people with AD can be regarded as either risk factors for AD, or conditions arising as a consequence of the developing pathological processes. The close relationship between pathological changes in AD and its comorbidities has been the subject of many studies. Understanding the mechanism of AD development and the influence of comorbidities on its pathogenesis may be crucial for creating an effective and complex pharmacotherapy for patients with AD Fig. 1.

### Comorbidities associated with age

The most relevant risk factor of Alzheimer's disease is age. AD mostly affects people above 65 years old, and so many patients also suffer from other diseases, such as diabetes mellitus type 2, hypercholesterolemia, atherosclerosis, and hypertension. It is well known that all the mentioned disorders are proved risk factors for AD, and it is supposed that they may trigger or accelerate Aβ and NFT formation and aggregation.

#### Diabetes mellitus type 2

Diabetes mellitus is a major health problem. It is estimated that 422 million people have diabetes worldwide, of whom, 90% suffer from diabetes mellitus type 2 (T2DM). The highest increase in the morbidity is observed in developed countries. It is believed that T2DM is currently one of the fastest growing chronic disorders worldwide [35].

T2DM is a chronic metabolic disorder characterized by persistent hyperglycemia caused by impaired insulin secretion or tissue insulin insensitivity to peripheral actions of insulin, or both [36]. Persistent hyperglycemia can cause organ failure, leading to the development of microvascular (retinopathy, nephropathy, and neuropathy) and macrovascular complications, increasing the risk of cardiovascular diseases. T2DM frequently coexists with other lifestyle diseases, such as hypercholesterolemia and hypertension [37]. Although some genetic factors are believed to exist, the main environmental risk factors are associated with an unhealthy lifestyle such as an unhealthy diet and physical inactivity leading to overweight and obesity [36, 37].

T2DM is a major risk factor for AD, and it is well documented that a high level of glucose and the presence of insulin resistance contribute to increased morbidity. Studies suggest that AD might be considered as the third type of diabetes [38]. As such, the relationship between AD and T2DM has been the subject of a number of studies. The insulin resistance associated with T2DM is thought to also play an essential role in the development of AD [39]. Not only does insulin maintain glucose homeostasis, affecting food intake, and glucose economy, it also protects neurons from degeneration, regulates the function of synapses, and directly influences the cognitive functions of the brain [36]. Insulin is also involved in β-amyloid production. The recent studies show that insulin can modulate the levels of β-amyloid by enhancing its production, and influencing the activity of BACE-1 and insulin-degrading enzyme (IDE), which degrades both insulin and β-amyloid [38]. Insulin resistance results in the maintenance of a high glucose level; such hyperglycemia in the course of T2DM results in the downregulation of insulin receptors in the brain and the accumulation of β-amyloid [38–41].

The main source of energy for the brain and neurons is glucose. Two transporters play a key role in glucose neuronal transport: GLUT1 (glucose transporter 1) and GLUT3 (glucose transporter 3). The recent studies suggest that insulin resistance might reduce the expression of GLUT3 and GLUT1 transporters on the neuronal membrane, as their localization and action are inversely related to glucose concentration; as such, insulin resistance may weaken neuronal glucose transport [42]. Such glucose transport disruption leads to impaired glucose metabolism, which results in the inhibition of choline acetyltransferase and a decreased level of acetylcholine in the synaptic cleft. Consequently, cholinesterase activity increases, which further decreases acetylcholine levels. This impairment of glucose uptake and metabolism is therefore regarded as a cause of neurodegeneration in AD [43].

The brain tissue of AD patients is characterized by significantly lower amounts of GLUT1 and GLUT3 transporters (25–30%) [42]. The expression of these two transporters is directly regulated by HIF-1 (hypoxia-inducible factor 1), which consists of two subunits: HIF-1α and HIF-1β. In AD, the expression of HIF-1, especially subunit α, is decreased [44], which may result in a decreased level of GLUT1 and GLUT3 transporters [42]. The cause of this downregulation is still not understood, although it is possible that it may be associated with the excessive oxidative stress and neuroinflammation accompanying AD [31]. In addition, impaired glucose uptake has been found to lead to hyperphosphorylation of tau protein by downregulation of tau O-GlcNAcylation [41]. Hypometabolism of glucose is considered an important factor in the pathogenesis of AD [41, 43].

Persistent hyperglycemia in the course of T2DM significantly increases the level of oxidative stress in the brain: the brains of AD patients are more susceptible to oxidative damage due to high oxygen levels and reduced antioxidants. Reactive-oxygen species activate neuroinflammation processes and accelerate β-amyloid and tau pathologies [45–47].

Since AD may be related to diabetes mellitus at the molecular level, in vitro/in vivo studies and clinical trials with drugs dedicated to T2DM could lead to promising results for future AD therapy [48]. Metformin, a biguanide used as an oral therapy for T2DM, has been shown in in vitro animal studies to increase Aβ clearance and reduce tau phosphorylation when given at doses significantly lower than used to treat type 2 diabetes mellitus. It is believed that the drug increases peripheral glucose uptake and regulates glucose metabolism by the activation of AMP-activated protein kinase (AMPK) [49]. However, the efficacy of metformin was proved only in an insulin-resistant neuron model [48, 49]. An ongoing clinical trial (phase II/phase III) of long-acting metformin in the prevention of Alzheimer’s disease, sponsored by Columbia University, (NCT04098666) may bring interesting results for future prevention strategy against AD.

Thiazolidinediones (TZDs), agonists of peroxisome proliferator-activated receptor γ (PPAR-γ), have also been advanced to clinical trials of AD [49]. Rosiglitazone and pioglitazone are used in the treatment of T2DM, resulting in reduced glucose levels and thus improved insulin sensitivity and lipid metabolism [49]. Moreover, TZDs also stimulate the expression of peroxisome proliferator-activated receptor-γ co-activator 1 α (PGC-1α) which participate in mitochondrial energy metabolism and antioxidant production [49]. PGC-1α expression has been found to be reduced in the brain tissue of patients with AD. First, a small clinical trial of pioglitazone on patients with mild-to-moderate AD revealed improvement in memory and cognition with reduced TNF-α [50]. However, no significant clinical efficacy was observed in phase II of a double-blind, placebo-controlled randomized controlled trial (NCT00982202) of pioglitazone in the therapy of AD [50]. A phase III clinical trial of rosiglitazone (NCT00550420), conducted after several promising studies with animal models of AD, has demonstrated some beneficial effects, such as reduced Aβ oligomers and inflammation with general cognitive improvement [51]. Unfortunately, the trial was terminated due to the results of the preliminary study and the presence of adverse effects, especially peripheral edema [51].

Several animal studies and clinical trials have examined the protective role of insulin against AD. In vitro and animal models have found insulin to be associated with increased Aβ oligomer clearance, decreased Aβ expression and various neuroprotective effects against Aβ [48, 49]. Enhanced insulin signaling protects synapses from binding by toxic Aβ oligomers; the stimulation of the insulin receptor may also mitigate such binding by downregulation [52]. Clinical trials with insulin have focused on the application of intranasal insulin in the treatment of mild AD and amnestic mild cognitive impairment (aMCI) [53]. This specific route of administration allows the bypass of the BBB; this is an important consideration as the transport of insulin to the brain occurs by an active transport mechanism which is often disrupted in the course of AD [53]. Moreover, the intranasal route significantly lowers the possibility of adverse effects in peripheral tissue [50, 54]. However, two clinical trials on 12-month treatment with intranasal Humulin® R U-100 (NCT01767909) and six-month treatment with intranasal glulisine (NCT02503501), completed in 2020, demonstrated no cognitive or functional benefits [53].

Liraglutide, a glucagon-like peptide-1 (GLP-1) analog, has also been evaluated for the use in the treatment of AD [49]. In vitro tests and animal studies of liraglutide revealed reduced levels of cerebral Aβ oligomers, normalized synaptic plasticity, and cerebral glucose uptake; it was also found to have neuroprotective and anti-inflammatory properties. Also, liraglutide appeared to stimulate neurogenesis [49]. A phase II (NCT01843075) clinical trial of liraglutide (ELAD study), currently ongoing, may indicate novel disease-modifying therapies for AD [55].

Many studies have confirmed a link between T2DM and AD [43, 56]. Hyperglycemia and insulin resistance accelerate brain damage in AD by inducing production and accumulation of amyloid β as well as enhancing hyperphosphorylation of tau protein [40], thus exacerbating the course of AD and its symptoms. It appears that control of the blood sugar level might be crucial in the therapy of AD.

#### Hypercholesterolemia and atherosclerosis

Hypercholesterolemia is a frequently observed disorder among seniors [57] which is exacerbated by genetic predispositions and environmental factors such as excessive lipid intake and sedentary lifestyle. The condition is an important risk factor in AD [58–61].

Several genes are related to hypercholesterolemia and the progression of AD. The most significant is the ApoE4 genotype, which contributes to the development of late-onset and sporadic AD [7]. ApoE is responsible for lipoprotein metabolism and the transport of cholesterol to neurons. Many studies indicate that ApoE4 promotes the aggregation of Aβ and the creation of neurofibrillary tangles [62, 63].

Hypercholesterolemia and the disruption of cholesterol metabolism play an important role in the pathology of AD, affecting Aβ formation, hyperphosphorylation of tau protein, disruption of cholinergic signaling, and neuroinflammation [63]. Under physiological conditions, cholesterol in the bloodstream is unable to cross the blood–brain barrier (BBB); hence, brain cholesterol is synthesized de novo by astrocytes and oligodendrocytes in situ [63]. In addition, essential polyunsaturated fatty acids (PUFAs) must be provided from food. An increased supply of PUFAs contributes to excessive brain synthesis of cholesterol, indicating that excessive dietary cholesterol intake directly influences the cholesterol economy of the brain [63–65].

The presence of increased cholesterol level in the brain has been found to enhance the permeability of the BBB [59]. Such dysfunction not only allows peripheral cholesterol to enter the brain but also other molecules that may damage the BBB. Hence, it appears that the proper function of the BBB might play an essential role in the progression of AD [63–65].

The mechanism of stimulation of Aβ production and accumulation by cholesterol has remains unknown. Cholesterol oxidation produces oxysterols, which play a role in the pathomechanism by crossing the BBB [63]. It is believed that oxysterols directly influence APP metabolism by influencing BACE-1 and γ-secretase to create toxic forms of β-amyloid [63]. Moreover, an increased level of oxysterols increases the formation of toxic, insoluble aggregates of Aβ from nontoxic soluble monomers [63–65].

An increased plasma cholesterol level also impairs cholinergic neurotransmission [64]. According to recent animal studies, the presence of an elevated level of oxysterols is thought to directly decrease the number of cholinergic neurons, which reduces acetylcholine levels in the brain [64]. In addition, an indirect pathway is also believed to exist; this results in the reduced activity of postsynaptic nicotinic and muscarinic receptors, as well as adrenergic receptors, which are capable of modulating the activity of cholinergic receptors [62–64].

The presence of an excessive number of oxysterols in the brain results in the production of high levels of oxidative stress and reactive oxygen species (ROS) which disrupt the oxidant–antioxidant balance [65]. This condition results in oxidative damage of cerebral tissue and activates microglia. These activated microglia contribute to chronic neuroinflammation by releasing cytokines and chemokines, such as tumor necrosis factor α (TNFα), interleukin 1 (IL-1), interleukin 6 (IL-6), and C-reactive protein (CRP) [64, 65].

Hypercholesterolemia clearly has a strong impact on the progression of AD, and maintaining cholesterol homeostasis is believed to play an important role in controlling the course of AD [62]. Currently, the results of studies of the use of statins in AD are inconsistent, and further research is needed [62, 63, 66].

Atherosclerosis is a disease that causes the deposition of plaques consisting of fat, cholesterol, or calcium among others, inside the arteries. This results in their walls thickening, which reduces the lumen and impairs the blood flow [67]. It has been suggested that intracranial and carotid atherosclerosis may be a possible factor in the pathomechanism of AD [68]. Vascular changes in the course of atherosclerosis result in hypoperfusion and hypoxia. Factors in plasma and from the endothelium stimulate arterial wall muscles to vasoconstrict, resulting in a reduction in blood flow [68]. This results in the secretion of hypoxia-inducible factor (HIF), which binds with the hypoxia-responsive element (HRE) on the BACE-1 promoter region and stimulates the expression of BACE-1 mRNA [68], thus causing excessive production of amyloid β and formation of plaques.

Chronic hypoperfusion, caused by atherosclerosis, leads to neuronal death, β-amyloid accumulation, and white matter damage [67], and the pathology of AD, especially the β-amyloid cascade, is known to promote intracranial atherosclerosis. In addition, the inflammation and oxidative stress generated in the course of atherosclerosis also hasten the course of AD [67]: pathological changes in the endothelium, such as vasoconstriction, reduce blood flow, and disrupt the blood supply to neurons, which impairs neuronal function [67, 68].

The ApoE4 genotype is a well known and documented risk factor for AD, and has a multidirectional impact on its pathogenesis [7]. ApoE4 causes dysregulation of lipids and lipoproteins, especially low-density lipoprotein (LDL) and cholesterol, and decreases glucose metabolism [6]. It also decreases Aβ clearance and reduces neuronal transmission [68]. ApoE4 also increases neuroinflammation and mitochondrial dysfunction, which enhances oxidative stress, blood–brain barrier breakdown and the leakage of blood-derived toxic proteins into the brain; it also reduces the length of small vessels, leading to neuronal damage [7]. Aβ accumulation and plaque formation are directly associated with the activity of ApoE4. Histopathological studies of brains of patients with AD show the presence of ApoE4 in β-amyloid plaques [6], and ApoE4 and Aβ have been found to share common site binding receptors that interact with each other [7]. In addition, ApoE4 is less effective in the clearance of Aβ than other isoforms, which significantly increases the risk of plaque formation and Aβ aggregation [6, 7, 68–74].

Statins represent the primary therapy used in the treatment of hypercholesterolemia and atherosclerosis [58]. Besides lowering cholesterol levels, statins offer a range of pleiotropic effects including beneficial antioxidant properties, improvement of endothelial function with increased nitric oxide bioavailability and anti-inflammatory properties, and stabilization of atherosclerotic plaques by reducing plaque lipids and thrombogenicity; these are believed to have a protective impact on dementia [58]. The positive influence of statins on dementia has been evaluated for decades in in vitro studies, animal models, and randomized trials; however, the results have been inconclusive [75–78]. It has been supposed that the ability of statins to reduce the risk of dementia is associated with their ability to cross the BBB and their molecular charge [77].

In vitro studies and those based on animal models indicate that hypercholesterolemia significantly increases Aβ deposition by promoting APP cleavage by γ- and β-secretase [49]. As statins effectively lower cholesterol levels, it was assumed that they may prevent dementia in long-term use. A randomized controlled trial in adult humans using simvastatin and pravastatin (NCT00939822) demonstrated an clear reduction of major vascular risk [75, 76]. However, an updated Cochrane review of double-blind, randomized, placebo-controlled trials where statins were administered for at least 12 months found them not to prevent dementia or cognitive decline in individuals at high vascular risk in late life [77, 78]. In contrast, a novel meta-analyses, including 55 observational studies from the last 20 years indicated that hydrophilic and lipophilic statins possess the potential to reduce dementia risk [79, 80].

#### Hypertension

Chronic hypertension is a major vascular risk factor contributing to dementia, including AD [81]. It affects one billion people worldwide. Recent studies have shown that controlling blood pressure (BP) in midlife significantly reduces the risk of cognitive impairment [81–83].

Long-term hypertension has a multidirectional influence on the development of AD. First, it contributes to intracranial and subcranial atherosclerosis, which results in lacunar infracts, micro-bleeding, white matter lesions and brain atrophy [84]. These lesions are directly responsible for increased metabolism of APP and decreased vascular clearance of Aβ [85]. One consequence of hypertension is brain hypoperfusion and hypoxia, resulting in impairment of cerebrovascular activity [86]. Disruption of cerebrovascular functions elevates oxidative stress, which impairs vessel dilation by reducing the production and bioavailability of nitric oxide (NO) and increasing that of the vasoconstriction factor endothelin 1 [84]. An imbalance between dilatory and constriction factors impairs cerebral blood flow and promotes thrombogenesis and atherosclerosis [84]. Hypertension causes stiffness of arteries and hypertrophic remodeling of vessels, which also leads to oxidative stress [83]. An excessive level of ROS causes neuroinflammation via the activation of microglia and production of cytokines, especially TNF-α and IL-6 [84]. These proinflammatory cytokines damage neurovascular coupling and deteriorate BBB functioning, which is related to β-amyloid deposition, formation of neurofibrillary tangles, and synaptic dysfunction [84–87].

Another important role in the development of AD associated with hypertension appears to be played by angiotensin II. Various studies indicate that angiotensin II influences plaque composition, and increased Aβ deposition via β- and γ-secretase and decreased Aβ clearance. Angiotensin II has also been directly implicated in brain hypoperfusion and cerebrovascular pathology [88].

Antihypertensive treatment becomes clinically relevant in the prophylaxis of AD [87]. Randomized trials have shown antihypertensive drugs to have a positive influence on the cognitive processes of patients with AD by exerting a protective effect on cerebral blood vessels [88]. This prevents ischemic damage to the grey matter regions essential for cognitive function [88, 89]. The Systolic Hypertension in Europe clinical trial evaluated the risk of vascular dementia in patients with diagnosed hypertension; the findings indicate that lowering blood pressure at least 20 mmHg below 150 mmHg reduces the incidence of dementia by 50% [78]. Several classes of antihypertensive drugs, e.g., calcium channel blockers (CCBs) and angiotensin-related compounds, have been considered as potential medication in AD [49]. Nilvadipine, a dihydropyridine calcium channel blocker, has been extensively studied for AD. In vitro and in vivo animal studies with nilvadipine revealed increased Aβ clearance, improved regional cerebral blood flow, and inhibited cerebral artery vasoconstriction [49]. Moreover, nilvadipine-treated mice demonstrated improved learning and memory abilities as compared to the nontreated group [49, 90, 91]. A large phase III clinical trial (NCT02017340) of nilvadipine as treatment for AD (NILVAD) revealed reduced blood pressure, improved cerebral blood flow in the hippocampus, and decreased cognitive decline. However, the findings suggest no benefits of nilvadipine as treatment of mild to moderate AD [92–94]. In vitro studies using the antihypertensive drug amlodipine according to in vitro studies inhibited Aβ production, but did not affect β-amyloid clearance [91]. The combination of amlodipine with losartan, atorvastatin, and exercise will be assessed in the phase III clinical trial: “Risk reduction for Alzheimer’s disease (rrAD)” (NCT02913664). This trial will evaluate the independent and cumulative effects of these pharmacological agents upon blood pressure, cholesterol control, and exercise training on neurocognitive function. The results of this clinical trial may have a significant impact on AD risk reduction [95].

Two classes of antihypertensive drugs related to angiotensin, viz*.* angiotensin-converting enzyme inhibitors (ACE-I) and angiotensin receptor blockers (ARBs), were proposed as candidates for decreasing incidents of all types of dementia [91]. Ramipril, an antihypertensive angiotensin-converting enzyme inhibitor was found to inhibit ACE activity in cerebrospinal fluid (CSF) and improve blood pressure in a recently-completed phase IV clinical trial (NCT00980785). However, ramipril at a dose of 5 mg/day did not influence Aβ1-42 level in CSF nor arterial function or cognition [96]. Epidemiological data found ARBs to demonstrate a stronger association with decreased dementia risk compared to ACE-I [97]. The ongoing phase II of SARTAN-AD clinical trial (NCT02085265) is designed to compare the effect of ACE-I perindopril with telmisartan, an angiotensin receptor blocker on brain atrophy in patients with mild to moderate AD. Also, a phase II double-blind placebo-control randomized clinical trial (NCT02646982) is also underway to evaluate the effect of candesartan on AD and related biomarkers. Future results will assess how blocking the effect of angiotensin II using candesartan affects cognitive functions in AD.

### Alzheimer's disease as a consequence of other diseases

#### Down’s syndrome

Down’s syndrome (DS) is a complex disorder arising from the trisomy of chromosome 21. It affects approximately 1 per 800 births [98, 99]. It was first described by John Langdon Down in 1866, and trisomy was later confirmed in 1959. The condition is a prevailing cause of mental retardation and a risk factor for AD. More than 50% of people with DS aged 40–50 years old develop AD [100], whereas this value is nearly 75% in the general population of people age above 60 [101–105].

The pathogenesis of the development of AD in people with DS includes β-amyloid deposition, tau hyperphosphorylation, neurofibrillary tangles, neuroinflammation, neuronal damage and loss, oxidative stress, and the disruption of synapses signaling [106]. The main cause of DS-dependent early-onset AD is the triplication of the *APP* genes lying on chromosome 21. Increased expression of *APP* gene leads to excessive production and accumulation of β-amyloid [107]. The disruption of the Aβ metabolism triggers other pathologies, including a deposition of neurofibrillary tangles and neuroinflammation [108]. The overexpression of *APP* gene also leads to increased release of cytokines, especially interleukin 1 (IL-1) and tumor necrosis factor α (TNF-α) [106]. The presence of high cytokine levels promotes neuroinflammation and microglial activation, which have a devastating influence on neurons and neuronal signaling [106, 107, 109–111].

It has been reported that in individuals with DS, mutation of several genes plays an important role in the development of AD [112]. One of them, *SOD1* (superoxide dismutase 1), is responsible for maintaining the oxidative balance. The overexpression of *SOD1* leads to an imbalance between oxidative factors and antioxidants, which contributes to neuroinflammation and increased Aβ production [112]. Also, the recent studies have shown that polymorphism of β-secretase (BACE-1) has a significant influence on the development of familial AD [113–115]. Mitochondrial dysfunction is frequently observed in people with DS. Disruption of mitochondrial processes, especially the mitochondrial respiratory chain, stimulates the production of ROS, which promotes the pathogenesis of AD and neuronal apoptosis. Mitochondrial dysfunction is believed to be directly responsible for cognitive decline because the resulting energy deficits disrupt cholinergic neurotransmission [98, 99, 112, 116–119]. Two cohort studies of AD in DS patients are currently recruiting participants (NCT04149197, NCT03901261), and this may shed light on the development of dementia in DS patients.

Metabolic studies using brain imaging with fluorodeoxyglucose positron emission tomography (FDG-PET) have revealed brain glucose hypometabolism in adults with DS and dementia [120]. Reduced glucose metabolism leads to enhanced Aβ and NFT accumulation, resulting in neuronal loss and synaptic dysfunction. The disruption of the glucose metabolism in the brain accelerates pathologies characteristic for AD [108, 120, 121].

Trisomy of chromosome 21 is associated with congenital disorders that may contribute to the development of AD. People with DS are frequently affected by congenital heart diseases, hypothyroidism, hyperlipidemia and hypercholesterolemia, obesity, and insulin resistance [119]. Lipid metabolism has a particularly strong impact on β-amyloid accumulation [112]. Individuals with DS have higher LDL, triglyceride (TG), total cholesterol, and lower high-density lipoprotein (HDL) levels as compared to those without DS, and this poses a risk factor of AD [116]. Interestingly, the recent studies found that Aβ production is correlated with plasma cholesterol level [122]. HDL has the ability to inhibit Aβ production and accumulation, and HDL directly influences the clearance of β-amyloid and can reduce oxidative stress [123]. Consequently, researchers have observed a link between a low HDL level and memory decline. However, studies on congenital disorders associated with DS and their impact on the development of AD should be continued [122–124].

### Comorbidities triggered by Alzheimer's disease

Neuropathological changes in the course of AD, including neurofibrillary tangles, Aβ deposits and brain atrophy, may simultaneously lead to the development of seizures, mental disorders, and sleep difficulties, which often affect people with AD. The occurrence of additional mental diseases accelerates and exacerbates the progression of dementia. Also, the occurrence of mental disorders in the course of AD significantly hinders successful therapy. Understanding the mechanism underlying the development of seizures, and mental and sleep disorders in the course of AD may provide information on how to treat and care for patients with AD.

#### Psychosis

Psychotic symptoms affect 40–60% of people with AD and mostly appear between the early and moderate stages of the disease [125]. The occurrence of neuropsychiatric symptoms distress patients and their caregivers and are often a reason for hospitalization. Moreover, studies have shown a correlation between psychosis in individuals with AD, increased mortality, and accelerated development of the disease [126]. Besides, the heritability of psychosis in AD and localized genes which is probably associated with AD and psychosis has been also confirmed [125–127].

Psychotic symptoms are defined by delusions, hallucinations, aggression, and agitation [128]. Delusions are manifested by infidelity, persecution, a sense of abandonment, and perceiving dead relatives as alive. Of various types of hallucinations, visual, and auditory hallucinations occur most frequently [129, 130]. Psychotic symptoms result from the neurodegeneration of the frontal lobes, visual cortex, and limbic regions of the brain [130]. Such damage is caused by many factors, particularly by excessive aggregation of β-amyloid and NFTs, which leads to atrophy [128]. Recent studies have demonstrated that phosphorylated tau protein plays a key role in women with psychosis in AD and α-synuclein in men. [129]. Although these gender differences have been clinically validated, the precise mechanism remains unclear. Even so, decreased cholinergic neurotransmission, significant reduction of a serotonin (5-HT) level, and increased dopaminergic neurotransmission have been linked with psychotic symptoms in patients with AD [128, 129, 131].

The treatment of psychosis in AD involves the administration of well-known antipsychotic drugs especially atypical antipsychotics, such as aripiprazole, olanzapine, quetiapine, and risperidone [128, 131]. Moreover, several animal models have shown atypical antipsychotics to have protective effects, including preventing apoptosis and enhancing neurogenesis in the dentate gyrus. In particular, olanzapine improved cognition by inhibiting the cholinergic receptor M2 and the serotonergic receptors 5HT3 and 5HT6 [49]. Unfortunately, a randomized controlled trial of olanzapine in patients with AD without evident psychobehavioral symptoms showed significant impairment of cognition as compared to placebo [132]. Similar results were obtained by clinical trial: “The CATIE-Alzheimer’s Disease Trial” (NCT00015548). Olanzapine, quetiapine, and risperidone, a second-generation antipsychotics, showed significant worsening of cognitive function as compared to placebo in multiple cognitive measurements over 36 weeks. Atypical antipsychotics exacerbate cognitive functions by blocking the dopamine D2 receptors in the mesocortical pathway, which connects the ventral tegmentum to the prefrontal cortex [133]. Therefore, the risk of cognitive impairment using atypical antipsychotics to treat patients with AD for psychobehavioral symptoms should be carefully considered [134].

#### Depression

Depression is one of the most frequent psychiatric disorders, being observed in nearly 50% of people with AD [135]. It has been confirmed that depression is directly correlated with the accelerated progression of AD. One of the characteristic features of AD is the neurodegeneration of the locus coeruleus (LC), the main norepinephrine (NE) nucleus in the brain [136]. The LC is vulnerable to tau pathology, and NFTs can be detected early before in other brain areas, often at the beginning of neurodegeneration [137]. As the LC plays an important role in the regulation of mood, attention, stress, motivation, and arousal, LC degeneration has been proposed as main cause of neuropsychiatric symptoms as depression, anxiety and sleep disorders [136]. It is believed that depression and AD follow similar neuropathological pathways [135]. The recent studies on mice indicate that injection of Aβ1-42 into the hippocampus leads to the occurrence of depressive-like symptoms; this has been associated with the occurrence of Aβ plaques and neurofibrillary tangles of tau protein in the hippocampus [138]. It has been proposed that the transneuronal spread of neurotoxic Aβ and tau into the limbic system triggers depression [138–140]. Another important cause of depressive behavior in patients with AD includes the presence of increased levels of glutamate and glutamine in the synaptic cleft [141]. A high level of glutamate is toxic for neurons and may lead to neuronal death [141]. Under physiological conditions in nerve terminals, glutamate is transported to the synaptic cleft by a vesicular glutamate transporter and released by exocytosis or directly from cytosol through plasma membrane proteins. The release of glutamate to the synaptic cleft is ATP and Ca^2+^ dependent. Extracellular glutamate binds to the receptor on the postsynaptic membrane of a neuron or is taken up by astrocytes. In an astroglial cell, glutamate is converted into glutamine by glutaminase; this is released to the extracellular compartment and then taken up by neurons and reconverted to glutamate inside the neuron. This glutamate–glutamine cycle is essential for preserving appropriate glutamate levels [142]. and its dysfunction may lead to an increased level of glutamate, resulting in neuronal failure [142, 143]. The exact mechanism of glutamate on the development of depression in patients with AD has not been fully clarified. However, it is hypothesized that a high level of glutamate observed in the hippocampus is crucial in the pathogenesis of depression in people with AD [141, 144]. In addition, other neurotransmitters also demonstrate disrupted levels in both depression and AD [144].

Changes in serotonergic neurotransmission play important roles in the pathomechanism of depression [145]. Patients with AD and with diagnosed depression demonstrate a decreased level of serotonin (5-HT) in the cortex and hippocampal 5-HT1A receptors [145]. In addition, studies have shown a significant decrease in dopamine and noradrenaline levels in patients diagnosed with AD and depression [144]. Cholinergic neurotransmission is also involved in the development of depression: a dramatically low level of acetylcholine (ACh) induces depression and anxiety in AD [144].

Neuroinflammation may be a cause of depression in AD [138]. Activated microglia produce proinflammatory cytokines such as interleukin 1β (IL-1β) and tumor necrosis factor α (TNF-α). Several studies have found high levels of IL-1β and TNF-α to be associated with depression symptoms and reduced plasticity of synapses [138].

The treatment of depression in AD patients often involves the use of selective serotonin reuptake inhibitors (SSRI) because they are well tolerated and have beneficial therapeutic effects [131, 146]. In animal models, SSRIs including citalopram, fluoxetine, paroxetine, and sertraline have shown to decrease Aβ levels, probably by inhibition of intracellular APP translation [145]. Moreover, chronic use of SSRI enhanced cognitive abilities, including hippocampal functions [145], this effect may be associated with the regulation of GSK-3β by serotonin [147]. Fluoxetine and paroxetine also increased brain-derived growth factor levels [49]. However, a clinical trial of citalopram (NCT00898807) in patients with probable AD and clinically significant agitation showed reduced agitation and caregiver distress [148]. However, the presence of serious cognitive and cardiac adverse effects limit citalopram in the therapy of psychobehavioral symptoms [148].

A well-known mood stabilizer, lithium carbonate, often used in the therapy of bipolar disorder has been suggested to have antidementia properties [149]. Lithium carbonate inhibits GSK-3β, which is associated with decreased tau phosphorylation [147]. According to studies performed in vitro and on animal models, lithium decreased β-amyloid production, tau phosphorylation, and prevented apoptosis by inhibiting membrane depolarization and cytochrome c release [49]. The results from the clinical trial (NCT01055392) with lithium as a disease-modifying agent in AD showed a significant decrease of phosphorylated tau and an increase of Aβ1-42 in CSF [149]. Moreover, long-term use of lithium weakens cognitive and functional impairment in patients with AD and amnestic mild cognitive impairment [149, 150]. Currently, ongoing clinical trials (NCT03185208) involving lithium as a possible treatment to prevent cognitive impairment in elders may elucidate the exact mechanism of lithium antidementia properties.

#### Sleep disorders

Sleep and circadian disturbances appear frequently in the course of AD. Approximately, 40% of people with AD have sleep disorders [151]. The most common being early morning awakenings, nighttime insomnia, daytime sleepiness, and napping during day time [152]. This sleep disturbance contributes to restlessness, confusion, and evening agitation in patients with AD and distresses their caregivers. Excessive daytime sleepiness may also lead to an increased probability of injury and hospitalization [151, 152].

Changes in sleep architecture and abnormalities in sleep–wake patterns are common in AD [152]. The most specific change is a quantitative reduction in the rapid eye movement stage (REM). Sleep disturbances have been associated with amyloid-β accumulation and neurofibrillary tangles influencing the secretion of neurotransmitters related to the sleep–wake cycle: hypocretins and melatonin [153]. Hypocretin 1 and 2 are secreted by the hypothalamus and are responsible for the stabilization of sleep–wake states and quality of sleep. β-amyloid accumulation directly decreases secretion of hypocretins, causing sleep disturbance [153]. Melatonin is a crucial hormone in the regulation of sleep in circadian rhythms [153]. It is produced in the pineal gland and has several physiological functions. Apart from the regulation of sleep–wake patterns, melatonin also supports the immune system and has an antioxidative effect. Also, melatonin has antiamyloidogenic properties; it regulates APP metabolism, reduces β-amyloid levels, and has antiaggregating properties [153]. A decreased level of melatonin in cerebrospinal fluid (CSF) can be seen as early as in the preclinical stage of AD. A low level of melatonin disrupts circadian rhythms, causing nighttime insomnia, daytime sleeping, and permanent restlessness and weakness [154]. The severity of sleep disturbance is directly correlated with the severity of cognitive impairment in AD [152–154].

Sleep disorders in patients with AD are typically treated with the administration of common drugs, such as melatonin, benzodiazepines, Z-hypnotics (zolpidem derivatives), sedating antidepressants, and antipsychotics [154].

Sleep-related breathing disorders (SRBDs) are also most frequent in patients with AD [153]. SRBDs cause hypoxia, which is responsible for cognitive impairment. Obstructive sleep apnea and hypopnea reduce the REM stage [154], causing frequent awakenings during nighttime. Nocturnal continuous positive airway pressure (CPAP) is the most successful treatment for sleep-related breathing disorders in patients with AD [153, 154].

#### Epilepsy

One of the most common neurological diseases whose incidence increases with age is epilepsy. According to World Health Organization (WHO) estimates, it affects 50 million people worldwide [155]. The most noticeable clinical manifestation is recurrent epileptic seizures, caused by an excessive and abnormal discharge of neurons, resulting in a depolarization wave called paroxysmal depolarizing shift [155].

An epileptic seizure is regarded as an important comorbidity of AD, with AD patients demonstrating a greater prevalence of epileptic episodes than non-AD individuals [156]. However, studies about epilepsy prevalence in AD individuals are not consistent and vary (1.5–64%) [157] depending on the study nature (prospective/retrospective) [158]. Epileptic seizures have been observed at the early stage of AD and MCI, but patients with advanced AD are exposed to higher epilepsy risk [159]. It seems that the male sex predisposes to a higher risk of epilepsy among AD patients. The exact relationship has not been elucidated yet, but it is probably related to the protective role of estrogens in epileptogenesis [155]. Moreover, it has been proposed that seizures may contribute to earlier cognitive decline and more rapid neurodegeneration progression [160].

Diagnosing epilepsy in individuals with dementia is challenging because confirmation of epilepsy especially, nonconvulsive focal seizure, may be difficult to differentiate from ordinary fluctuations in the course of AD [161]. Periodic worsening of the condition in patients with AD, associated with hallucinations, confusion, consciousness, and attention disturbance, can mask focal, nonconvulsive seizures [160]. Moreover, epilepsy diagnosis requires confirmation of a history of epileptic seizures, which is difficult to achieve in demented patients. According to International League Against Epilepsy (ILAE) guidelines, epilepsy can be confirmed if the patient has had one or two unrelated epileptic seizures, but EEG (electroencephalography) must show interictal epileptiform discharges (IEDs) [162]. In AD patients, IEDs are mostly localized in the temporal lobe and frontotemporal area, and its prevalence in AD individuals ranges from 20 to 60% [155]. Several studies have tried to evaluate the dominant type of seizure in AD [155]. According to the results, both focal and generalized seizure occurs in AD patients. However, focal seizures appear to dominate, regardless of the secondary generalization [155]. Focal seizures mostly affect the temporal lobe and limbic structure, i.e., the hippocampus [155, 159].

Among patients with AD, the burden of epilepsy development is significantly higher for those with the inherited form of AD, which is associated with autosomal-dominant mutation of specific genes encoding the APP protein, PSEN1 and PSEN2 [158]. The prevalence of epilepsy was 60% in patients with APP duplication and 45% for those with PSEN1 mutation. Mutation of PSEN2 has a lower risk of epilepsy (about 30%). In case of Down’s Syndrome, where three copies of the APP gene exist due to trisomy, epilepsy is observed in 84% of cases [155, 157].

The link between AD and epilepsy is complex and involves various mechanisms. Epilepsy and AD are considered to share similar pathological hallmarks and processes, e.g., Aβ accumulation, tau protein, GSK3β, and neuroinflammation, which mutually affect each other and promote aggravation of the pathologies [155].

Aβ deposition has been proposed as an important factor in epileptogenesis in patients with AD [163]. The exact mechanism has not been fully elucidated. Aβ has multidimensional effects on the synapses, neuronal circuits, and neuronal transmission. Several human APP mutant AD mouse models [163, 164] indicate that pathological Aβ levels induce neuronal hyperexcitability, manifested by nonconvulsive epileptiform activity in the hippocampus, cortical areas, and abnormal membrane depolarization in cortical pyramidal cells [158, 163, 164]. Moreover, excessive levels of Aβ impair synaptic plasticity and disrupt excitatory synaptic transmission [157]. The compensation mechanisms activated in response to epileptic seizures may influence normal synaptic and neuronal functions in cortical and limbic areas of the brain which are crucial for learning and memory [159]. This proposed mechanism, involving Aβ, may be a link between epilepsy and cognitive impairment [155]. A 2018 observational, prospective study about the possible role of amyloid β pathology in AD, and late-onset epilepsy of unknown origin (LOEU) [165] showed a significant relationship between late-onset epilepsy and CSF Aβ with progression to AD [166]. Moreover, pathological levels of Aβ_1-42_ in CSF in patients with stable cognitive functions suggest that Aβ_1-42_ may be an important link between AD neurodegeneration and epileptogenesis [165]. Cognitive functions in patients with late-onset epilepsy should be carefully monitored to provide quick diagnosis and treatment.

Pathological tau protein accumulation and NFTs have been implicated in cases of epilepsy in individuals with AD [167]. Since hyperphosphorylated tau protein has been observed in patients with diagnosed epilepsy, several mechanisms have been proposed to elucidate the role of tau in epileptogenesis [167]. The hypothesis that hyperphosphorylated tau is related to neuronal hyperexcitability [159] has been confirmed in an animal model using antisense oligonucleotides [158]. Reduced tau levels exert protective effects on cognitive function and epileptiform activity. In addition, hyperphosphorylated tau protein induces significant abnormalities in neuronal connectivity and synaptic plasticity, resulting in a higher seizure risk [168]. Studies based on the overexpressing human mutated tau mice revealed a significant increase in glutamate level and dysfunction of NMDA receptors (NMDAR), resulting in excitotoxicity [155]. NMDAR overactivation promotes neuronal damage and excessive tau phosphorylation which contribute to cognitive impairment [160, 167]. The mechanisms linking tau pathology with epilepsy and AD are still the subjects of extensive study.

The proline-directed kinase GSK3β may play a role epileptogenesis and neuronal hyperexcitability [167]. GSK3β is located in the axons of the CNS and is mainly responsible for tau phosphorylation. Increased GSK3β activity results in abnormal tau phosphorylation and NFT accumulation [167]. Moreover, GSK3β can modulate cellular apoptosis pathways and decrease the levels of transcription factors, such as HSF1 (heat shock transcription factor 1) which modulate the cellular response to stress. Several studies have demonstrated a relationship between overexpression and activation of GSK3β, and cognitive impairment [167]. Animal tests with lithium [168], a well-known GSK3β inhibitor, revealed a significant reduction of phosphorylated tau levels and neuronal damage prevention, as well as an improvement in cognitive functions in rats, including memory and spatial learning. The role of GSK3β in epileptogenesis results from its physiological functions, and is strongly related to tau protein activity [168]. Although GSK3β overexpression does not cause epilepsy, it significantly increases the neuronal susceptibility to seizures [167]. Consequently, GSK3β should be considered as an important part of the complex mechanism linking epilepsy and AD.

Neuroinflammation is an important factor in developing neurodegenerative disorders. Several studies indicate that neuroinflammation plays a role in seizure development [158]. Generally, neuroinflammation includes an initiated immune response in CNS including activation of astrocytes and microglia, releasing specific endothelial cells, cytokines, and signaling molecules. Neuroinflammation can be induced by various factors, i.e., brain injury, CNS infections, neurodegenerative disorders, autoimmune processes, and toxic substances. Epileptic seizures may be caused by neuroinflammation, and pro-inflammatory factors can often lead to seizures in patients with epilepsy [158]. Studies with patients affected by temporal lobe epilepsy using PET revealed many inflammatory markers compared to healthy individuals [155]. Animal studies have shown that prolonged and recurrent seizures provoke glia activation and abnormal production of prostaglandins, cytokines, such as IL-1β, TNF-α, IL-6, and growth factors [155]. This may result in the disruption of the BBB and the involvement of peripheral immune cells. Moreover, released cytokines may upregulate activation of NMDA receptors, resulting in excessive intracellular calcium influx leading to neuronal damage or apoptosis. Consequently, cytokines especially IL-1β, may contribute to the development of epilepsy by inducing an imbalance between inhibitory and excitatory factors [155]. The activated microglia are also believed to play a role in seizure development by inhibiting neurogenesis [155]. The important role played by neuroinflammation in developing both epilepsy and AD is supported by evidence from animal and human studies [158].

The treatment of epileptic seizures in patients with AD should be adjusted to the individual, taking into consideration the seizures reduction without cognitive aggravation [169]. Unfortunately, many antileptic drugs (AEDs) worsen cognitive functions in AD patients [158]. Generally, the first class of AEDs including, phenobarbital and phenytoin should be avoided due to their influence on cognitive functions and on sleepiness and osteomalacia [158]. Valproic acid and benzodiazepines are also associated with cognitive impairment. Carbamazepine also is associated with many adverse effects including arrhythmia, hyponatremia, and decrease of bone marrow functions. Gabapentin is safe from the pharmacological point of view because it shows no significant interactions [158]; however, it may be ineffective in advanced AD. Levetiracetam and lamotrigine present improvements in neuropsychological symptoms and epileptic seizures [158, 169]. Moreover, the benefits of levetiracetam include a broad spectrum of action, linear pharmacokinetics, renal secretion, and slight improvement in cognitive function [159]. Two clinical phase II (NCT04004702; NCT03489044) trials will evaluate the effects of levetiracetam in patients with AD and neuropsychiatric symptoms related to epilepsy. The results from these studies may confirm the beneficial effect of levetiracetam on AD individuals and clarify the molecular mechanisms linking epilepsy and other neuropsychiatric symptoms with AD.

## Conclusion

The present paper presents the most common groups of comorbidities associated with AD: those related to age, those triggered by AD and those associated with Down’s syndrome. Age is the most relevant risk factor for AD [3]. Hence, age-related comorbidities, such as hypertension, type 2 diabetes mellitus (T2DM), atherosclerosis, and hypercholesterolemia are most common in patients with AD. All studies provide information about the potential influence of these disorders on the pathogenesis of AD, particularly on Aβ and tau pathologies [33].

Since AD is considered the third type of diabetes mellitus, it is essential to maintain proper glucose levels [37]. Medications used in the therapy of T2DM have been repurposed for AD therapy. In vitro and in vivo studies of several classes of drugs used in T2DM, viz*.* metformin, thiazolidinediones, intranasal insulin, and GLP-1 analogs, improved cognitive function and reduced AD biomarkers in CSF [49, 50]. However, clinical trials revealed no significant improvements in cognition or reduced progression in patients with mild to moderate AD and aMCI [51, 54, 55]. Nevertheless, ongoing clinical trials of long-acting metformin in the prevention of AD (NCT04098666) may provide interesting results on how long-term control of glucose levels can influence dementia progression.

Chronic hypertension is an important risk factor for dementia. Consequently, blood pressure control becomes relevant to the prevention and treatment of AD [81]. Several classes of antihypertensive medications have been studied as repurposing agents in the treatment of AD [49]. After very promising in vivo studies, nilvadipine, a representative of CCBs, was evaluated in NILVAD clinical trial (NCT02017340) [94]. Despite beneficial partial results, including improved hippocampal cerebral blood flow, reduced blood pressure, and decreased cognitive decline, final results exposed no clinically relevant benefits. The novel, ongoing clinical trial (NCT02913664), where patients will be treated with a combination of amlodipine, losartan, atorvastatin, and exercise may reveal important information on how blood pressure, cholesterol, and physical activity influence risk reduction of dementia.

Hypercholesterolemia has been implicated in increased risk for dementia and AD [59]. Statins are the main class of drugs used for treatment of hypercholesterolemia and atherosclerosis due to their ability to lower cholesterol levels and their pleiotropic effects, due to which, statins were proposed as repurposing medications in the treatment of AD [77]. However, clinical trials of dementia risk reduction by statins returned inconclusive results [75, 76]. Nevertheless, a meta-analysis of over 50 observational studies from the last two decades indicates that statins offer beneficial effects in dementia risk reduction [80].

Adults with Down’s syndrome are at high risk of developing AD [107]. The progression of pathological changes characteristic for AD associated with triplication of the APP gene and the disorders accompanying Down’s syndrome, require deeply thought decisions concerning pharmacotherapy and care [101].

Comorbidities triggered by AD are also important factors in therapy. The occurrence of psychobehavioral symptoms and epileptic seizures in the course of AD significantly complicates therapy and accelerates disease progression [126, 159]. It is vital to establish a proper neurological and psychiatric diagnosis with quick initiating treatment control the progression of AD and provide a better quality of life for patients with AD [125]. The treatment of psychotic episodes in AD should be carefully considered and monitored because atypical antipsychotics may result in cognitive impairment [131].

In conclusion, the research strongly suggests that comorbidities have an important influence on AD pathologies. Although the relationship between AD and its comorbidities remains unclear, there is growing awareness and concern about this issue. Even so, these conditions are still treated as independent disorders. Successful therapy of AD requires a new approach to its comorbidities and implementation of complex polypharmacotherapy.

## References

[1] Patterson C. World Alzheimer Report 2018. The State of the Art of Dementia Research: New Frontiers. London, UK: Alzheimer’s disease International; 2018. https://www.alz.co.uk/research/WorldAlzheimerReport2018.pdf.

[2] Prince M, Wimo A, Guerchet M, Ali GC, Wu YT, Prina M. World Alzheimer Report 2015. The Global Impact of Dementia: An Analysis of Prevalence, Incidence, Cost and Trends. London: Alzheimer’s disease International; 2015. https://www.alz.co.uk/research/WorldAlzheimerReport2015.pdf.

[3] Prince M, Guerchet MM, Prina M. The Epidemiology and Impact of Dementia: Current State and Future Trends. WHO Thematic Briefing. 2015. https://www.who.int/mental_health/neurology/dementia/dementia_thematicbrief_epidemiology.pdf.

[4] Jack CR, Bennett DA, Blennow K, Carrillo MC, Dunn B, Haeberlein SB (2018). NIA-AA Research Framework: Toward a biological definition of Alzheimer's disease. Alzheimers Dement.

[5] Bajda M, Jończyk J, Malawska B, Czarnecka K, Girek M, Olszewska P (2015). Synthesis, biological evaluation and molecular modeling of new tetrahydroacridine derivatives as potential multifunctional agents for the treatment of Alzheimer’s disease. Bioorg Med Chem.

[6] Dorszewska J, Prendecki M, Oczkowska A, Dezor M, Kozubski W (2016). Molecular basis of familial and sporadic Alzheimer's disease. Curr Alzheimer Res.

[7] Prendecki M, Florczak-Wyspianska J, Kowalska M, Ilkowski J, Grzelak T, Bialas K (2019). APOE genetic variants and apoE, miR-107 and miR-650 levels in Alzheimer's disease. Folia Neuropathol.

[8] Ungar L, Altmann A, Greicius MD (2014). Apolipoprotein E, gender, and Alzheimer's disease: an overlooked, but potent and promising interaction. Brain Imaging Behav.

[9] Saxena M, Dubey R (2019). Target enzyme in Alzheimer's disease: acetylcholinesterase inhibitors. Curr Top Med Chem.

[10] Verma S, Kumar A, Tripathi T, Kumar A (2018). Muscarinic and nicotinic acetylcholine receptor agonists: current scenario in Alzheimer's disease therapy. J Pharm Pharmacol.

[11] Szymański P, Markowicz M, Mikiciuk-Olasik E (2011). Synthesis and biological activity of derivatives of tetrahydroacridine as acetylcholinesterase inhibitors. Bioorg Chem.

[12] Wang XL, Xiong Y, Yang Y, Tuo QZ, Wang XC, Chen R (2015). A novel tacrine-dihydropyridine hybrid (-)SCR1693 induces tau dephosphorylation and inhibits Abeta generation in cells. Eur J Pharmacol.

[13] Keri RS, Quintanova C, Chaves S, Silva DF, Cardoso SM, Santos MA (2016). New tacrine hybrids with natural-based cysteine derivatives as multitargeted drugs for potential treatment of Alzheimer's disease. Chem Biol Drug Des.

[14] Mao F, Li J, Wei H, Huang L, Li X (2015). Tacrine-propargylamine derivatives with improved acetylcholinesterase inhibitory activity and lower hepatotoxicity as a potential lead compound for the treatment of Alzheimer's disease. J Enzyme Inhib Med Chem.

[15] Nepovimova E, Korabecny J, Dolezal R, Babkova K, Ondrejicek A, Jun D (2015). Tacrine-trolox hybrids: a novel class of centrally active, nonhepatotoxic multi-target-directed ligands exerting anticholinesterase and antioxidant activities with low in vivo toxicity. J Med Chem.

[16] Chen Y, Liu Z, Fu TM, Li W, Xu X, Sun HP (2015). Discovery of new acetylcholinesterase inhibitors with small core structures through shape-based virtual screening. Bioorg Med Chem Lett..

[17] Horak M, Holubova K, Nepovimova E, Krusek J, Kaniakova M, Korabecny J (2017). The pharmacology of tacrine at N-methyl-d-aspartate receptors. Prog Neuropsychopharmacol Biol Psychiatry.

[18] Arndt JW, Qian F, Smith BA, Quan C, Kilambi KP, Bush MW (2018). Structural and kinetic basis for the selectivity of aducanumab for aggregated forms of amyloid-beta. Sci Rep.

[19] Sevigny J, Chiao P, Bussiere T, Weinreb PH, Williams L, Maier M (2016). The antibody aducanumab reduces Abeta plaques in Alzheimer's disease. Nature.

[20] Graham WV, Bonito-Oliva A, Sakmar TP (2017). Update on Alzheimer's disease therapy and prevention strategies. Annu Rev Med.

[21] Flores E, Peña-Ortega F. Amyloid β Peptide-Induced Changes in Prefrontal Cortex Activity and Its Response to Hippocampal Input. Int J Pept.2017;1–9. 10.1155/2017/7386809 PMC5239987 28127312

[22] Vaz M, Silvestre S (2020). Alzheimer's disease: Recent treatment strategies. Eur J Pharmacol.

[23] Briggs R, Kennelly SP, O'Neill D (2016). Drug treatments in Alzheimer's disease. Clin Med (Lond).

[24] Kabir MT, Uddin MS, Mamun AA, Jeandet P, Aleya L, Mansouri RA (2020). Combination drug therapy for the management of Alzheimer's disease. Int J Mol Sci.

[25] Maramai S, Benchekroun M, Gabr MT, Yahiaoui S (2020). Multitarget therapeutic strategies for Alzheimer's disease: review on emerging target combinations. Biomed Res Int.

[26] Hunter S, Brayne C (2017). Do anti-amyloid beta protein antibody cross reactivities confound Alzheimer disease research?. J Negat Results Biomed..

[27] Oh H, Steffener J, Razlighi QR, Habeck C, Stern Y (2016). β-Amyloid deposition is associated with decreased right prefrontal activation during task switching among cognitively normal elderly. J Neurosci.

[28] Zeng H, Wu X (2016). Alzheimer's disease drug development based on computer-aided drug design. Eur J Med Chem.

[29] Hu SQ, Wang R, Cui W, Mak SH, Li G, Hu YJ (2015). Dimeric bis (heptyl)-cognitin blocks Alzheimer's beta-amyloid neurotoxicity via the inhibition of abeta fibrils formation and disaggregation of preformed fibrils. CNS Neurosci Ther.

[30] Boutajangout A, Wisniewski T (2014). Tau-based therapeutic approaches for Alzheimer's disease - a mini-review. Gerontology.

[31] Obulesu M, Jhansilakshmi M (2014). Neuroinflammation in Alzheimer's disease: an understanding of physiology and pathology. Int J Neurosci.

[32] Haaksma ML, Vilela LR, Marengoni A, Calderon-Larranaga A, Leoutsakos JMS, Rikkert M (2017). Comorbidity and progression of late onset Alzheimer's disease: A systematic review. PLoS ONE.

[33] Santiago JA, Potashkin JA (2021). The impact of disease comorbidities in Alzheimer's disease. Front Aging Neurosci..

[34] Bernardes C, Massano J, Freitas A (2018). Hospital admissions 2000–2014: a retrospective analysis of 288 096 events in patients with dementia. Arch Gerontol Geriatr.

[35] Guariguata L, Whiting DR, Hambleton I, Beagley J, Linnenkamp U, Shaw JE (2014). Global estimates of diabetes prevalence for 2013 and projections for 2035. Diabetes Res Clin Pract.

[36] Chornenkyy Y, Wang WX, Wei A, Nelson PT (2019). Alzheimer's disease and type 2 diabetes mellitus are distinct diseases with potential overlapping metabolic dysfunction upstream of observed cognitive decline. Brain Pathol.

[37] Chentli F, Azzoug S, Mahgoun S (2015). Diabetes mellitus in elderly. Indian J Endocrinol Metab.

[38] Yang HT, Sheen YJ, Kao CD, Chang CA, Hu YC, Lin JL (2013). Association between the characteristics of metabolic syndrome and Alzheimer's disease. Metab Brain Dis.

[39] De Felice FG, Lourenco MV, Ferreira ST (2014). How does brain insulin resistance develop in Alzheimer's disease?. Alzheimers Dement.

[40] Paul KC, Jerrett M, Ritz B (2018). Type 2 diabetes mellitus and Alzheimer's disease: overlapping biologic mechanisms and environmental risk factors. Curr Environ Health Rep.

[41] Zhu Y, Shan X, Yuzwa SA, Vocadlo DJ (2014). The emerging link between O-GlcNAc and Alzheimer disease. J Biol Chem.

[42] Szablewski L. Glucose Transporters in Brain: In Health and in Alzheimer's Disease. Alzheimers Dis. 2017;55(4):1307–20. 10.3233/JAD-160841 27858715

[43] de Nazareth AM (2017). Type 2 diabetes mellitus in the pathophysiology of Alzheimer's disease. Dement Neuropsychol.

[44] Ashok BS, Ajith TA, Sivanesan S (2017). Hypoxia-inducible factors as neuroprotective agent in Alzheimer's disease. Clin Exp Pharmacol Physiol.

[45] Karki R, Kodamullil AT, Hofmann-Apitius M (2017). Comorbidity analysis between Alzheimer's disease and type 2 diabetes mellitus (T2DM) based on shared pathways and the role of T2DM drugs. J Alzheimers Dis.

[46] Ogama N, Sakurai T, Kawashima S, Tanikawa T, Tokuda H, Satake S (2018). Postprandial hyperglycemia is associated with white matter hyperintensity and brain atrophy in older patients with type 2 diabetes mellitus. Front Aging Neurosci.

[47] Girard H, Potvin O, Nugent S, Dallaire-Theroux C, Cunnane S, Duchesne S (2018). Faster progression from MCI to probable AD for carriers of a single-nucleotide polymorphism associated with type 2 diabetes. Neurobiol Aging..

[48] Nday CM, Eleftheriadou D, Jackson G (2018). Shared pathological pathways of Alzheimer's disease with specific comorbidities: current perspectives and interventions. J Neurochem.

[49] Appleby BS, Nacopoulos D, Milano N, Zhong K, Cummings JL (2013). A review: treatment of Alzheimer's disease discovered in repurposed agents. Dement Geriatr Cogn Disord.

[50] Folch J, Petrov D, Ettcheto M, Abad S, Sanchez-Lopez E, Garcia ML (2016). Current research therapeutic strategies for Alzheimer's disease treatment. Neural Plast.

[51] Gold M, Alderton C, Zvartau-Hind M, Egginton S, Saunders AM, Irizarry M (2010). Rosiglitazone monotherapy in mild-to-moderate Alzheimer's disease: results from a randomized, double-blind, placebo-controlled phase III study. Dement Geriatr Cogn Disord.

[52] Smith LM, Strittmatter SM (2017). binding sites for amyloid-beta oligomers and synaptic toxicity. Cold Spring Harb Perspect Med.

[53] Craft S, Raman R, Chow TW, Rafii MS, Sun CK, Rissman RA (2020). Safety, efficacy, and feasibility of intranasal insulin for the treatment of mild cognitive impairment and Alzheimer disease dementia a randomized clinical trial. JAMA Neurol.

[54] Yiannopoulou KG, Papageorgiou SG (2020). Current and future treatments in Alzheimer disease: an update. J Cent Nerv Syst Dis.

[55] Femminella GD, Frangou E, Love SB, Busza G, Holmes C, Ritchie C (2019). Evaluating the effects of the novel GLP-1 analogue liraglutide in Alzheimer's disease: study protocol for a randomised controlled trial (ELAD study). Trials.

[56] Sun Y, Ma C, Sun H, Wang H, Peng W, Zhou Z (2020). Metabolism: a novel shared link between diabetes mellitus and Alzheimer's disease. J Diabetes Res.

[57] Farzadfar F, Finucane MM, Danaei G, Pelizzari PM, Cowan MJ, Paciorek CJ (2011). National, regional, and global trends in serum total cholesterol since 1980: systematic analysis of health examination surveys and epidemiological studies with 321 country-years and 3.0 million participants. Lancet..

[58] Dias HK, Brown CL, Polidori MC, Lip GY, Griffiths HR (2015). LDL-lipids from patients with hypercholesterolaemia and Alzheimer's disease are inflammatory to microvascular endothelial cells: mitigation by statin intervention. Clin Sci (Lond).

[59] Wood WG, Li L, Muller WE, Eckert GP (2014). Cholesterol as a causative factor in Alzheimer's disease: a debatable hypothesis. J Neurochem.

[60] Bahety P, Van Nguyen TH, Hong Y, Zhang L, Chan ECY, Ee PLR (2017). Understanding the cholesterol metabolism-perturbing effects of docosahexaenoic acid by gas chromatography-mass spectrometry targeted metabonomic profiling. Eur J Nutr.

[61] Cheng Y, Jin Y, Unverzagt FW, Su L, Yang L, Ma F (2014). The relationship between cholesterol and cognitive function is homocysteine-dependent. Clin Interv Aging.

[62] Ricciarelli R, Canepa E, Marengo B, Marinari UM, Poli G, Pronzato MA (2012). Cholesterol and Alzheimer's disease: a still poorly understood correlation. IUBMB Life.

[63] Xue-Shan Z, Juan P, Qi W, Zhong R, Li-Hong P, Zhi-Han T (2016). Imbalanced cholesterol metabolism in Alzheimer's disease. Clin Chim Acta.

[64] Ullrich C, Pirchl M, Humpel C (2010). Hypercholesterolemia in rats impairs the cholinergic system and leads to memory deficits. Mol Cell Neurosci.

[65] Ettcheto M, Petrov D, Pedros I, de Lemos L, Pallas M, Alegret M (2015). Hypercholesterolemia and neurodegeneration. Comparison of hippocampal phenotypes in LDLr knockout and APPswe/PS1dE9 mice. Exp Gerontol..

[66] Whitmer RA, Gunderson EP, Quesenberry CP, Zhou J, Yaffe K (2007). Body mass index in midlife and risk of Alzheimer disease and vascular dementia. Curr Alzheimer Res.

[67] Kalaria RN, Akinyemi R, Ihara M (2012). Does vascular pathology contribute to Alzheimer changes?. J Neurol Sci.

[68] Arslan A, Tuzun FA, Arslan H, Demir H, Tamer S, Demir C (2016). The relationship between serum paraoxonase levels and carotid atherosclerotic plaque formation in Alzheimer's patients. Neurol Neurochir Pol.

[69] Yarchoan M, Xie SX, Kling MA, Toledo JB, Wolk DA, Lee EB (2012). Cerebrovascular atherosclerosis correlates with Alzheimer pathology in neurodegenerative dementias. Brain.

[70] Wendell CR, Waldstein SR, Ferrucci L, O'Brien RJ, Strait JB, Zonderman AB (2012). Carotid atherosclerosis and prospective risk of dementia. Stroke.

[71] Gutierrez J, Honig L, Elkind MS, Mohr JP, Goldman J, Dwork AJ (2016). Brain arterial aging and its relationship to Alzheimer dementia. Neurology.

[72] Dolan H, Crain B, Troncoso J, Resnick SM, Zonderman AB, Obrien RJ (2010). Atherosclerosis, dementia, and Alzheimer disease in the baltimore longitudinal study of aging cohort. Ann Neurol.

[73] Stamatelopoulos K, Sibbing D, Rallidis LS, Georgiopoulos G, Stakos D, Braun S (2015). Amyloid-beta (1–40) and the risk of death from cardiovascular causes in patients with coronary heart disease. J Am Coll Cardiol.

[74] Holtzman DM (2001). Role of apoe/Abeta interactions in the pathogenesis of Alzheimer's disease and cerebral amyloid angiopathy. J Mol Neurosci.

[75] Shepherd J, Blauw GJ, Murphy MB, Bollen E, Buckley BM, Cobbe SM (2002). Pravastatin in elderly individuals at risk of vascular disease (PROSPER): a randomised controlled trial. Lancet.

[76] Collins R, Armitage J, Parish S, Sleight P, Peto R (2002). Heart protection study C. MRC/BHF heart protection study of cholesterol lowering with simvastatin in 20536 high-risk individuals: a randomised placebo-controlled trial. Lancet..

[77] McGuinness B, Craig D, Bullock R, Passmore P (2016). Statins for the prevention of dementia. Cochrane Database Syst Rev.

[78] Cao JQ, Hou JW, Ping J, Cai DM (2018). Advances in developing novel therapeutic strategies for Alzheimer's disease. Mol Neurodegener.

[79] Poly TN, Islam M, Walther BA, Yang HC, Wu CC, Lin MC (2020). Association between use of statin and risk of dementia: a meta-analysis of observational studies. Neuroepidemiology.

[80] Chu CS, Tseng PT, Stubbs B, Chen TY, Tang CH, Li DJ (2018). Use of statins and the risk of dementia and mild cognitive impairment: A systematic review and meta-analysis. Sci Rep.

[81] Gabin JM, Tambs K, Saltvedt I, Sund E, Holmen J (2017). Association between blood pressure and Alzheimer disease measured up to 27 years prior to diagnosis: the HUNT Study. Alzheimers Res Ther.

[82] Viswanathan A, Greenberg SM, Scheltens P (2016). Role of vascular disease in Alzheimer-like progressive cognitive impairment. Stroke.

[83] Vemuri P, Lesnick TG, Przybelski SA, Knopman DS, Lowe VJ, Graff-Radford J (2017). Age, vascular health, and Alzheimer disease biomarkers in an elderly sample. Ann Neurol.

[84] Thorin E (2015). Hypertension and Alzheimer disease: another brick in the wall of awareness. Hypertension.

[85] Nagai M, Hoshide S, Kario K (2010). Hypertension and dementia. Am J Hypertens.

[86] Hamel E, Royea J, Ongali B, Tong XK (2016). Neurovascular and cognitive failure in Alzheimer's disease: benefits of cardiovascular therapy. Cell Mol Neurobiol.

[87] Marfany A, Sierra C, Camafort M, Domenech M, Coca A (2018). High blood pressure, Alzheimer disease and antihypertensive treatment. Panminerva Med.

[88] Iadecola C, Yaffe K, Biller J, Bratzke LC, Faraci FM, Gorelick PB (2016). Impact of hypertension on cognitive function: a scientific statement from the american heart association. Hypertension.

[89] Lawlor B, Segurado R (2018). Nilvadipine in mild to moderate Alzheimer disease: A randomised controlled trial. PLoS Med..

[90] Paris D, Quadros A, Humphrey J, Patel N, Crescentini R, Crawford F (2004). Nilvadipine antagonizes both A beta vasoactivity in isolated arteries, and the reduced cerebral blood flow in APPsw transgenic mice. Brain Res.

[91] Paris D, Bachmeier C, Patel N, Quadros A, Volmar CH, Laporte V (2011). Selective antihypertensive dihydropyridines lower a beta accumulation by targeting both the production and the clearance of a beta across the blood-brain barrier. Mol Med.

[92] Lawlor B, Segurado R, Kennelly S, Rikkert M, Howard R, Pasquier F (2018). Nilvadipine in mild to moderate Alzheimer disease: A randomised controlled trial. PLoS Med.

[93] de Jong DLK, de Heus RAA, Rijpma A, Donders R, Rikkert M, Gunther M (2019). Effects of nilvadipine on cerebral blood flow in patients with Alzheimer disease a randomized trial. Hypertension.

[94] Abdullah L, Crawford F, Tsolaki M, Borjesson-Hanson A, Rikkert MO, Pasquier F (2020). The influence of baseline Alzheimer's disease severity on cognitive decline and CSF biomarkers in the NILVAD trial. Front Neurol.

[95] Szabo-Reed AN, Vidoni E, Binder EF, Burns J, Cullum CM, Gahan WP (2019). Rationale and methods for a multicenter clinical trial assessing exercise and intensive vascular risk reduction in preventing dementia (rrAD Study). Contemp Clin Trials.

[96] Wharton W, Stein JH, Korcarz C, Sachs J, Olson SR, Zetterberg H (2012). The effects of ramipril in individuals at risk for Alzheimer's disease: results of a pilot clinical trial. J Alzheimers Dis.

[97] Davies NM, Kehoe PG, Ben-Shlomo Y, Martin RM (2011). Associations of anti-hypertensive treatments with Alzheimer's disease, vascular dementia, and other dementias. J Alzheimers Dis.

[98] Wiseman FK, Al-Janabi T, Hardy J, Karmiloff-Smith A, Nizetic D, Tybulewicz VL (2015). A genetic cause of Alzheimer disease: mechanistic insights from Down syndrome. Nat Rev Neurosci.

[99] Barone E, Head E, Butterfield DA, Perluigi M (2017). HNE-modified proteins in Down syndrome: involvement in development of Alzheimer disease neuropathology. Free Radic Biol Med.

[100] Mahla RS (2019). Down syndrome cognitive marker's significance in Alzheimer's disease and dementia management. Alzheimers Dement.

[101] Butterfield DA, Perluigi M (2018). Down syndrome: From development to adult life to Alzheimer disease. Free Radic Biol Med.

[102] Lin AL, Powell D, Caban-Holt A, Jicha G, Robertson W, Gold BT (2016). (1)H-MRS metabolites in adults with Down syndrome: Effects of dementia. Neuroimage Clin.

[103] Chen XQ, Sawa M, Mobley WC (2018). Dysregulation of neurotrophin signaling in the pathogenesis of Alzheimer disease and of Alzheimer disease in down syndrome. Free Radic Biol Med.

[104] Sauna-Aho O, Bjelogrlic-Laakso N (2018). Signs indicating dementia in Down, williams and fragile X syndromes. Mol Genet Genomic Med.

[105] Cenini G, Fiorini A, Sultana R, Perluigi M, Cai J, Klein JB (2014). An investigation of the molecular mechanisms engaged before and after the development of Alzheimer disease neuropathology in Down syndrome: a proteomics approach. Free Radic Biol Med.

[106] Cohen AD, McDade E, Christian B, Price J, Mathis C, Klunk W (2018). Early striatal amyloid deposition distinguishes Down syndrome and autosomal dominant Alzheimer's disease from late-onset amyloid deposition. Alzheimers Dement.

[107] Head E, Powell D, Gold BT, Schmitt FA (2012). Alzheimer's disease in Down syndrome. Eur J Neurodegener Dis.

[108] Milenkovic I, Jarc J, Dassler E, Aronica E, Iyer A, Adle-Biassette H (2018). The physiological phosphorylation of tau is critically changed in fetal brains of individuals with Down syndrome. Neuropathol Appl Neurobiol.

[109] Ovchinnikov DA, Korn O, Virshup I, Wells CA, Wolvetang EJ (2018). The impact of APP on Alzheimer-like pathogenesis and gene expression in Down syndrome iPSC-derived neurons. Stem Cell Reports.

[110] Hamlett ED, Boger HA, Ledreux A, Kelley CM, Mufson EJ, Falangola MF (2016). Cognitive impairment, neuroimaging, and Alzheimer neuropathology in mouse models of Down syndrome. Curr Alzheimer Res.

[111] Fortea J, Carmona-Iragui M, Benejam B, Fernandez S, Videla L, Barroeta I (2018). Plasma and CSF biomarkers for the diagnosis of Alzheimer's disease in adults with Down syndrome: a cross-sectional study. Lancet Neurol.

[112] Montoya JC, Fajardo D, Pena A, Sanchez A, Dominguez MC, Satizabal JM (2014). Global differential expression of genes located in the Down syndrome critical region in normal human brain. Colomb Med.

[113] Maia MA, Sousa E (2019). BACE-1 and γ-secretase as therapeutic targets for Alzheimer's disease. Pharmaceuticals (Basel).

[114] Rampa A, Gobbi S, Concetta Di Martino RM, Belluti F, Bisi A (2017). Dual BACE-1/GSK-3beta inhibitors to combat Alzheimer's disease: a focused review. Curr Top Med Chem..

[115] Sun J, Roy S (2018). The physical approximation of APP and BACE-1: A key event in Alzheimer's disease pathogenesis. Dev Neurobiol.

[116] Wilcock DM, Griffin WS (2013). Down's syndrome, neuroinflammation, and Alzheimer neuropathogenesis. J Neuroinflammation.

[117] Perluigi M, Butterfield DA (2012). Oxidative stress and Down syndrome: a route toward alzheimer-like dementia. Curr Gerontol Geriatr Res..

[118] Malakooti N, Pritchard MA, Adlard PA, Finkelstein DI (2014). Role of metal ions in the cognitive decline of Down syndrome. Front Aging Neurosci.

[119] Klosowska A, Cwiklinska A, Kuchta A, Berlinska A, Jankowski M, Wierzba J (2017). Down syndrome, increased risk of dementia and lipid disturbances. Dev Period Med.

[120] Head E, Powell DK, Schmitt FA (2018). Metabolic and vascular imaging biomarkers in Down syndrome provide unique insights into brain aging and alzheimer disease pathogenesis. Front Aging Neurosci.

[121] Head E, Phelan MJ, Doran E, Kim RC, Poon WW, Schmitt FA (2017). Cerebrovascular pathology in Down syndrome and Alzheimer disease. Acta Neuropathol Commun.

[122] Choong XY, Tosh JL, Pulford LJ, Fisher EM (2015). Dissecting Alzheimer disease in Down syndrome using mouse models. Front Behav Neurosci.

[123] Lanfranchi S, Jerman O, Dal Pont E, Alberti A, Vianello R (2010). Executive function in adolescents with Down syndrome. J Intellect Disabil Res.

[124] Gholipour T, Mitchell S, Sarkis RA, Chemali Z (2017). The clinical and neurobehavioral course of Down syndrome and dementia with or without new-onset epilepsy. Epilepsy Behav.

[125] Weamer EA, DeMichele-Sweet MA, Cloonan YK, Lopez OL, Sweet RA (2016). Incident psychosis in subjects with mild cognitive impairment or Alzheimer's disease. J Clin Psychiatry.

[126] Storti LB, Quintino DT, Silva NM, Kusumota L, Marques S (2016). Neuropsychiatric symptoms of the elderly with Alzheimer's disease and the family caregivers' distress. Rev Lat Am Enfermagem..

[127] Devanand DP, Pelton GH, D'Antonio K, Strickler JG, Kreisl WC, Noble J (2017). Low-dose lithium treatment for agitation and psychosis in Alzheimer disease and frontotemporal dementia: a case series. Alzheimer Dis Assoc Disord.

[128] Shah C, DeMichele-Sweet MA, Sweet RA (2017). Genetics of psychosis of Alzheimer disease. Am J Med Genet B Neuropsychiatr Genet.

[129] DeMichele-Sweet MAA, Weamer EA, Klei L, Vrana DT (2018). Genetic risk for schizophrenia and psychosis in Alzheimer disease. Mol Psychiatry.

[130] Koppel J, Sousa A, Gordon ML, Giliberto L, Christen E, Davies P (2018). Association between psychosis in elderly patients with Alzheimer disease and impaired social cognition. JAMA Psychiat.

[131] Koppel J, Greenwald BS (2014). Optimal treatment of Alzheimer's disease psychosis: challenges and solutions. Neuropsychiatr Dis Treat.

[132] Kennedy J, Deberdt W, Siegal A, Micca J, Degenhardt E, Ahl J (2005). Olanzapine does not enhance cognition in non-agitated and non-psychotic patients with mild to moderate Alzheimer's dementia. Int J Geriatr Psychiatry.

[133] De Deyn PP, Drenth AFJ, Kremer BP, Voshaar RCO, Van Dam D (2013). Aripiprazole in the treatment of Alzheimer's disease. Expert Opin Pharmacother.

[134] Vigen CLP, Mack WJ, Keefe RSE, Sano M, Sultzer DL, Stroup TS (2011). Cognitive Effects of atypical antipsychotic medications in patients with Alzheimer's disease: outcomes from CATIE-AD. Am J Psychiatry.

[135] Mess E, Witkowicz M, Ornat M, Sielski P, Klaszczyk T (2018). Evaluation of depression in patients with Alzheimer's disease according to the location of medical care. Arch Psychiatr Nurs.

[136] Weinshenker D (2018). Long road to ruin: noradrenergic dysfunction in neurodegenerative disease. Trends Neurosci.

[137] Matchett BJ, Grinberg LT, Theofilas P, Murray ME (2021). The mechanistic link between selective vulnerability of the locus coeruleus and neurodegeneration in Alzheimer's disease. Acta Neuropathol.

[138] Amani M, Shokouhi G, Salari AA (2019). Minocycline prevents the development of depression-like behavior and hippocampal inflammation in a rat model of Alzheimer's disease. Psychopharmacology (Berl)..

[139] Zhang Q-E, Ling S, Li P, Zhang S, Ng CH, Ungvari GS (2018). The association between urinary Alzheimer-associated neuronal thread protein and cognitive impairment in late-life depression: a controlled pilot study. Int J Biol Sci.

[140] Schipke CG, De Vos A, Fuentes M, Jacobs D, Vanmechelen E, Peters O (2018). Neurogranin and BACE1 in CSF as potential biomarkers differentiating depression with cognitive deficits from early Alzheimer's disease: a pilot study. Dement Geriatr Cogn Dis Extra.

[141] Madeira C, Vargas-Lopes C, Brandão CO, Reis T, Laks J, Panizzutti R (2018). Elevated glutamate and glutamine levels in the Cerebrospinal fluid of patients with probable Alzheimer's disease and depression. Front Psychiatry.

[142] Danbolt NC (2001). Glutamate uptake. Prog Neurobiol.

[143] Wang R, Reddy PH (2017). Role of glutamate and NMDA receptors in Alzheimer's disease. J Alzheimers Dis.

[144] Xu H, Wang Z, Zhu L, Sui Z, Bi W, Liu R (2018). Targeted neurotransmitters profiling identifies metabolic signatures in rat brain by LC-MS/MS: application in insomnia, depression and Alzheimer's disease. Molecules.

[145] Mdawar B, Ghossoub E, Khoury R (2020). Selective serotonin reuptake inhibitors and Alzheimer's disease. Neural Regen Res.

[146] Van Dam D, Vermeiren Y, Dekker AD, Naude PJ, Deyn PP. Neuropsychiatric Disturbances in Alzheimer's Disease: What Have We Learned from Neuropathological Studies? Curr Alzheimer Res. 2016;13(10):1145–64. 10.2174/1567205013666160502123607 PMC5070416 27137218

[147] Kisby B, Jarrell JT, Agar ME, Cohen DS, Rosin ER, Cahill CM, et al. Alzheimer's disease and its potential alternative therapeutics. J Alzheimers Dis Parkinsonism. 2019;9(5). 10.4172/2161-0460.1000477 PMC6777730 31588368

[148] Porsteinsson AP, Drye LT, Pollock BG, Devanand DP, Frangakis C, Ismail Z (2014). Effect of citalopram on agitation in Alzheimer disease The CitAD randomized clinical trial. JAMA.

[149] Forlenza OV, Diniz BS, Radanovic M, Santos FS, Talib LL, Gattaz WF (2011). Disease-modifying properties of long-term lithium treatment for amnestic mild cognitive impairment: randomised controlled trial. Br J Psychiatry.

[150] Forlenza OV, Radanovic M, Talib LL, Gattaz WF (2019). Clinical and biological effects of long-term lithium treatment in older adults with amnestic mild cognitive impairment: randomised clinical trial. Br J Psychiatry.

[151] Camargos EF, Pandolfi MB, Dias MP, Quintas JL, Guimaraes RM, Nobrega OT (2011). Incidence of sleep disorders in patients with Alzheimer disease. Einstein (Sao Paulo).

[152] Bombois S, Derambure P, Pasquier F, Monaca C (2010). Sleep disorders in aging and dementia. J Nutr Health Aging.

[153] Ju YS, Videnovic A, Vaughn BV. Comorbid Sleep Disturbances in Neurologic Disorders. Continuum (Minneap Minn). 2017;23(4, Sleep Neurology):1117–31. 10.1212/CON.0000000000000501 28777179

[154] Urrestarazu E, Iriarte J (2016). Clinical management of sleep disturbances in Alzheimer's disease: current and emerging strategies. Nat Sci Sleep.

[155] Giorgi FS, Saccaro LF, Busceti CL, Biagioni F, Fornai F (2020). Epilepsy and Alzheimer's disease: potential mechanisms for an association. Brain Res Bull.

[156] Rauramaa T, Saxlin A, Lohvansuu K, Alafuzoff I, Pitkänen A, Soininen H (2018). Epilepsy in neuropathologically verified Alzheimer's disease. Seizure.

[157] Friedman D, Honig LS, Scarmeas N (2012). Seizures and epilepsy in Alzheimer's disease. CNS Neurosci Ther.

[158] Powell G, Ziso B, Larner AJ (2019). The overlap between epilepsy and Alzheimer's disease and the consequences for treatment. Expert Rev Neurother.

[159] Miranda DDC, Brucki SMD (2014). Epilepsy in patients with Alzheimer's disease: A systematic review. Dement Neuropsychol.

[160] Paudel YN, Angelopoulou E, Jones NC, O'Brien TJ, Kwan P, Piperi C (2019). Tau Related pathways as a connecting link between epilepsy and Alzheimer's disease. ACS Chem Neurosci.

[161] Nicastro N, Assal F, Seeck M (2016). From here to epilepsy: the risk of seizure in patients with Alzheimer's disease. Epileptic Disord.

[162] Horváth A, Szűcs A, Hidasi Z, Csukly G, Barcs G, Kamondi A (2018). Prevalence, semiology, and risk factors of epilepsy in Alzheimer's disease: an ambulatory EEG study. J Alzheimers Dis.

[163] Minkeviciene R, Rheims S, Dobszay MB, Zilberter M, Hartikainen J, Fülöp L (2009). Amyloid beta-induced neuronal hyperexcitability triggers progressive epilepsy. J Neurosci.

[164] Palop JJ, Mucke L (2009). Epilepsy and cognitive impairments in Alzheimer disease. Arch Neurol.

[165] Costa C, Romoli M, Liguori C, Farotti L, Eusebi P, Bedetti C (2019). Alzheimer's disease and late-onset epilepsy of unknown origin: two faces of beta amyloid pathology. Neurobiol Aging.

[166] Costa C, Parnetti L, D'Amelio M, Tozzi A, Tantucci M, Romigi A (2016). Epilepsy, amyloid-β, and D1 dopamine receptors: a possible pathogenetic link?. Neurobiol Aging.

[167] Toral-Rios D, Pichardo-Rojas PS, Alonso-Vanegas M, Campos-Peña V (2020). GSK3β and tau protein in Alzheimer's disease and epilepsy. Front Cell Neurosci.

[168] Garg N, Joshi R, Medhi B (2018). Cracking novel shared targets between epilepsy and Alzheimer's disease: need of the hour. Rev Neurosci.

[169] Cretin B (2018). Pharmacotherapeutic strategies for treating epilepsy in patients with Alzheimer's disease. Expert Opin Pharmacother.

